# A Derived Positional Mapping of Inhibitory Subtypes in the Somatosensory Cortex

**DOI:** 10.3389/fnana.2019.00078

**Published:** 2019-08-06

**Authors:** Daniel Keller, Julie Meystre, Rahul V. Veettil, Olivier Burri, Romain Guiet, Felix Schürmann, Henry Markram

**Affiliations:** ^1^Blue Brain Project, École Polytechnique Fédérale de Lausanne, Geneva, Switzerland; ^2^Laboratory of Neural Microcircuitry, École Polytechnique Fédérale de Lausanne, Lausanne, Switzerland; ^3^Faculty of Health Sciences, Ben-Gurion University of the Negev, Beer Sheva, Israel; ^4^Bioimaging and Optics Platform, École Polytechnique Fédérale de Lausanne, Lausanne, Switzerland

**Keywords:** cell density, rat brain, inhibitory interneurons, somatosensory cortex, composition, cell types, cell counting, neuronal distribution

## Abstract

Obtaining a catalog of cell types is a fundamental building block for understanding the brain. The ideal classification of cell-types is based on the profile of molecules expressed by a cell, in particular, the profile of genes expressed. One strategy is, therefore, to obtain as many single-cell transcriptomes as possible and isolate clusters of neurons with similar gene expression profiles. In this study, we explored an alternative strategy. We explored whether cell-types can be algorithmically derived by combining protein tissue stains with transcript expression profiles. We developed an algorithm that aims to distribute cell-types in the different layers of somatosensory cortex of the developing rat constrained by the tissue- and cellular level data. We found that the spatial distribution of major inhibitory cell types can be approximated using the available data. The result is a depth-wise atlas of inhibitory cell-types of the rat somatosensory cortex. In principle, any data that constrains what can occur in a particular part of the brain can also strongly constrain the derivation of cell-types. This draft inhibitory cell-type mapping is therefore dynamic and can iteratively converge towards the ground truth as further data is integrated.

## Introduction

Mapping the anatomical location of interneurons subtypes within the cerebral cortex is an unsolved problem. Interneurons exhibit different morphological and electrophysiological properties and consequently, each type of interneuron plays a unique role in nervous system function (Markram et al., 2004). Therefore, properly placing cells in the correct layer is an important step in creating models of cortical function. Simulations of the cortex, for example, can potentially use the resulting cell type-specific densities (Markram et al., 2015; Schmidt et al., 2018).

The definition of what constitutes particular cell types is not yet fully established, though standards are emerging (Petilla Interneuron Nomenclature Group, 2008). Neurons in the cortex can be classified in terms of their electrical and morphological properties, the projection patterns, and the proteins and genes they express (ibid.). We expect that the most detailed classification will come from single-cell transcriptomes. As many as 50 cortical interneuron types may exist (Lim et al., 2018). A definitive classification together with a distribution approach will allow establishment of the cell-type composition of the whole brain, brain regions, areas and nuclei, and layers in any region (Zeisel et al., 2015).

Currently, inhibitory neurons in rodent somatosensory cortex can be categorized into at least the following morphological types: Martinotti cells (MC), Double Bouquet cells (DB), Bitufted Cells (BC), Bipolar cells (BP), Neurogliaform Cells (NGC), Chandelier Cells (ChC), Small Basket Cells (SBC), Large Basket Cells (LBC), and Nest Basket Cells (NBC; Markram et al., 2004). Additional sub-classification can be made according to electrophysiology (Contreras, 2004), and their transcript expression (Zeisel et al., 2015; Lake et al., 2016; Mi et al., 2018).

Here, we develop a fitting approach to first establish the distribution of morphologically defined inhibitory cell types based on their molecular staining. We focus on interneurons because there is a clearly-identified subset of proteins known to be expressed in separate populations of interneurons: calbindin (CB), calretinin (CR), neuropeptide Y (NPY), parvalbumin (PV), somatostatin (SOM) and vasointestinal peptide (VIP; Rudy et al., 2011; Tremblay et al., 2012). These protein markers have been previously shown as extremely valuable tools for the classification and identification of inhibitory neuron subtypes (Xu et al., 2010). The fitting process then adjusts the density of the inhibitory neuron types in order to best match the density of markers seen in multiple experimentally stained tissue sections.

Based on these approaches, morphological interneuron types can then be categorized depending on the expression level (RNA and/or protein) of each of these markers. For example, NGC cells are positive for NPY (Zambrano, 2012), ChC cells are positive for PV (Tremblay et al., 2016) and in some cases CB (del Rio and DeFelipe, 1997; Rocco et al., 2017), whereas BTC cells can be positive for all markers except PV (Arbib, 2003). In this study, we use cell counts obtained from fluorescent immunohistochemistry images of these proteins in conjunction with reverse-transcriptase polymerase chain reaction (RT-PCR) measurements of transcript expression levels as cell type makers, to predict the number of each inhibitory cell morphology type, through the depth of the somatosensory cortex of P14 rat.

We provide an algorithm to distribute cellular types in the cortical column of the developing rat that matches well the experimental data. The presented method improves biological relevance of digital simulations of the neocortex, and provides a framework for the improvement of models of other brain regions and cell subtypes as more data becomes available. For example, single-cell transcriptomics data for many genes is now available (Zeisel et al., 2015, 2018; Tasic et al., 2016, 2018; Saunders et al., 2018), though co-registration with electrophysiological and morphological properties is not always performed.

## Materials and Methods

### Immunohistochemistry

#### Animals

All animal procedures were approved by the Veterinary Authorities and the Cantonal Commission for Animal Experimentation of the Canton of Vaud, according to the Swiss animal protection laws.

Outbred Wistar Han rats (Janvier Laboratories, France) were ordered with their litter aged to 8 postnatal days (P8). Dams were housed individually and allowed to raise their own litters until experimentation on male offspring on P14. Animals were housed in standard plastic laboratory cages, with bedding, nesting material and paper tube and *ad libitum* access to food and water, cleaned once a week, and kept in a 12 h light-dark schedule with lights on at 6:30 am, in rooms under controlled humidity and temperature.

#### Brain Tissue Processing

On P14, rats were transferred to the experimental room and allowed to acclimatize, before being deeply anesthetized with pentobarbital (intraperitoneal dose 150 mg/kg; conc. 150 mg/ml), followed by transcardial perfusion with cold 0.1 M phosphate buffer (PB; pH 7.4), then by cold 4% paraformaldehyde (PFA) in PB 0.1 M. The brain was dissected from the skull, postfixed overnight in 4% PFA (4°C) and rinsed in PB 0.1 M. Brains were consecutively stored in 15% sucrose solution (in PB 0.1 M) at 4°C during an approximate of 24 h, followed by 30% sucrose solution at 4°C during approximately 24 h. Sagittal sections from the right hemisphere were cut on a cryostat at 50 μm with an approximate angle of 3–5 degree rotation along the anterior-posterior axis. Brain slices were stored in cryoprotectant until immunohistochemistry assays.

#### Protein Staining

**Figure 1:**
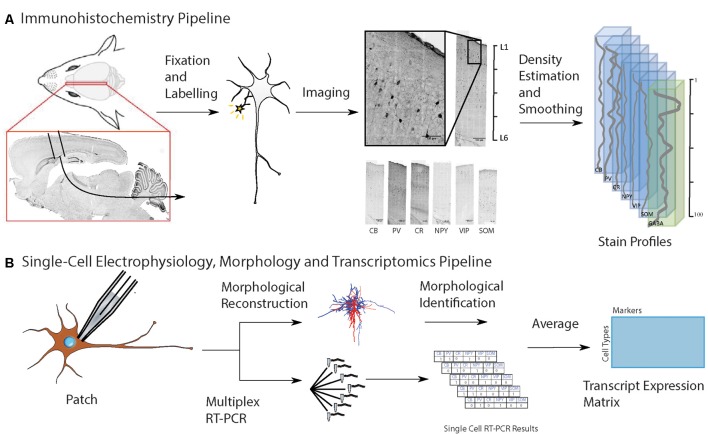
Workflow for input data to predict cell-type distribution profiles. **(A)** Experimental pipeline to obtain anatomical location of interneuron subtypes based on immunostaining profiles: first, collect and fix brain tissue, then run separate immunoassays on separate brain slices, each staining for a known cell type-specific protein, then take the average distribution of each cell type, and combine them on a map of the cortical column. Six different inhibitory cell markers were stained for, as well as neuronal nuclear protein (NeuN) and γ-aminobutyric acid (GABA). **(B)** Experimental pipeline to obtain interneuron single-cell morphology and transcriptomics: harvest tissue and patch-clamp to record the electrophysiological profile while injecting biocytin intracellularly, followed by cytoplasmic harvesting (Toledo-Rodriguez et al., 2005). Subsequent tissue fixation and digital reconstruction give cell morphology. Reverse-transcriptase polymerase chain reaction (RT-PCR) of the harvested cytoplasm yields transcript expression levels. The RT-PCR transcript expression data is averaged on a cell type-specific basis to produce a refined transcript expression matrix. The entries correspond to the proportion of cells of a particular morphological type expressing a particular marker, so the rows and columns do not need to add up to unity.

To quantify the density of the total neurons in the neocortical column, we first stained the brain slice using antibodies against neuronal nuclear protein (NeuN); which is expressed in all neurons; and γ-aminobutyric acid (GABA); which is specific to inhibitory interneurons (methods and results detailed in Markram et al., 2015; Figure 1A). The primary antibodies were mouse anti-neuron specific nuclear protein 1:1,000 (anti-NeuN, Chemicon, MAB377) and rabbit anti-GABA 1:500 (anti-GABA, Sigma-Aldrich Inc., A2052).

We re-counted the original dataset and mapped the resulting density into 100 equal-width bins extending from the top of layer 1 (L1) to the bottom of layer 6 (L6). The obtained cell densities were smoothed using a moving average of width four bins. The smooth curves were rescaled to match counts obtained through stereology in the center of each layer (Markram et al., 2015). The scaling factor differed between layers and was interpolated at points between the layer centers in order to obtain a contiguous output. Smoothing was done by fitting the averaged trace with a piecewise linear function with line segments of width five bins and averaging the results of using all possible starting offsets. Inhibitory interneuron density was also measured for use as a later constraint in the fitting process.

To obtain the layer-dependent protein expression data in P14 rat, brain slices from somatosensory cortex (from Bregma: 1.90 to 2.40 mm lateral; Paxinos and Watson, 1998) were immunostained and processed against inhibitory cell protein markers (Table 1) following previously-published methods (Markram et al., 2015), using the following proteins: CB, CR, NPY, PV, SOM and VIP. For visualization of laminar and area boundaries and for cell counting quality procedures, slices were stained for 4′,6-diamidino-2-phenylindole (DAPI; Figure 1A, Supplementary Table S1).

**Table 1:** Antibodies in use for each marker.

	Primary antibody	Secondary antibody	Nucleic acid staining
Calbindin (CB)	Mouse monoclonal anti-calbindin, Swant 300, 1:2,500	Donkey anti-mouse, Alexa Fluor 568, Invitrogen A10037, 1:1,000	DAPI, Sigma-Aldrich D9542, 1:25,000
Calretinin (CR)	Mouse monoclonal anti-calretinin, Swant 6B3, 1:5,000	Donkey anti-mouse, Alexa Fluor 568, Invitrogen A10037, 1:1,000	DAPI, Sigma-Aldrich D9542, 1:25,000
Neuropeptide Y (NPY)	Rabbit polyclonal anti-neuropeptide Y, Immunostar 22940, 1 :2,500	Donkey anti-rabbit, Alexa Fluor 568, Invitrogen A10042, 1:1,000	DAPI, Sigma-Aldrich D9542, 1:25,000
Parvalbumin (PV)	Goat polyclonal anti-parvalbumin, Swant PVG-213, 1:2,000	Donkey anti-goat, Alexa Fluor 568, Invitrogen A11057, 1:1,000	DAPI, Sigma-Aldrich D9542, 1:25,000
Somatostain-14 (SOM)	Rabbit polyclonal anti-somatostatin, Peninsula T-4103, 1:2,500	Donkey anti-rabbit, Alexa Fluor 568, Invitrogen A10042, 1:1,000	DAPI, Sigma-Aldrich D9542, 1:25,000
Vasointestinal peptide (VIP)	Rabbit polyclonal anti-vasoactive intestinal peptide, Immunostar 20077, 1:750	Donkey anti-rabbit, Alexa Fluor 568, Invitrogen A10042, 1:1,000	DAPI, Sigma-Aldrich D9542, 1:25,000

#### Microscopes and Immunofluorescence (IF)

Standard confocal microscopy was performed on multi-color immunostained brain slices on a confocal microscope (LSM700, Zeiss) in the upright configuration with 40×/1.30 NA Plan-Apochromat oil-immersion objective (Zeiss). Acquisitions of the neocortical column on its entire length and slice thickness (50 μm) were performed with a zoom factor of 1 (to minimize uneven illumination artifacts), with a pixel size of 0.15 μm and a z-step of 1 μm, and with pinhole size set at 33 μm (or 1.0 Airy unit, optimized for A568), leading to an optical section of 1.0 μm. The DAPI signal was obtained using laser excitation at 405 nm; single-probe-labeled slices were excited with 555 nm laser. Images were visualized using ZEN software (ZEN 2009, Zeiss) and processed using the open source image processing package Fiji (Schindelin et al., 2012; public domain, GPL v2 license). A quality check of brain regions was performed on images captured with a slide scanner (Olympus, VS120-L100) with a 10×/0.40 UPLSAPO air objective (Olympus) by visually comparing them with the Rat Brain Anatomy Atlas (Paxinos and Watson, 2014). Data were excluded if not part of the primary somatosensory cortex, hindlimb region (S1HL).

#### Image Processing

Image processing (stitching, reslicing, maximum intensity projections and height map calculation), the cell counts and the layer boundaries drawings were performed with Fiji processing package.

#### Cell Counting

Cells immunostained for the expression of specific protein were counted following stereology rules on the resliced projections, using the cell counter plugin from Fiji^1^. The whole S1HL acquired volume was counted. The top and left borders were excluded from the cell counts but we have considered the –z top and the –z bottom of the slice being part of the volume. A slice thickness of 50 μm (equal to the theoretical slicing thickness) was used in the volume calculation. Cell counts were divided by layer volume to obtain an estimate for the density of cells expressing a particular marker within each bin.

To facilitate counting of cells, a series of macros were used to relate the cursor’s position between two opened images (one of which is assumed to be a reslice from the other). A cell was considered being positive when both the DAPI and the specific cell marker were stained. DAPI co-staining was necessary to ensure that cell bodies and not finer processes were counted. Cells that were doubtful or negative for one or both the conditions were excluded. Cell counts were performed by one experimenter and peer-verified completely at least once to ensure that: (1) no cells were missing; (2) each cell marked was positive for the marker and DAPI; and (3) no cells were counted twice.

### Single-Cell Electrophysiology, Morphology and Transcriptomics

We used a data set obtained in a previously-published work (Toledo-Rodriguez et al., 2005). Individual neurons in P13–P16 rat somatosensory cortex were characterized for their electrical, morphological and transcript expression (RNA) properties. Briefly, during whole-cell patch-clamp electrophysiological recordings, neurons were also loaded intracellularly with the chemical compound biocytin, for subsequent immunohistochemistry and digital reconstruction of their morphological characteristics. To obtain cell-type transcript expression profiles, the same cells with electrophysiological measurements and biocytin injected for morphological reconstructions, also had their cytoplasm harvested at the end of experimentation, for single-cell RT-PCR measurements. The same six proteins targeted in the immunohistochemistry experiment were studied (CB, CR, PV, NPY, VIP and SOM). This allowed characterization of cells according to morphological types (Figure 1B). For each cell, the detected RNA expression of a target gene was encoded as one and its absence as zero. For a given cell, this resulted in a binary vector whose length was the number of genes. Multiple instances of each morphological cell type were present in the complete data set.

### Fitting Procedure

**Figure 2:**
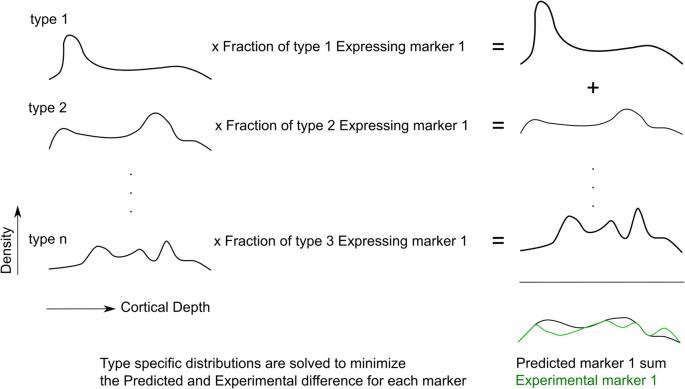
The fitting procedure. Cell type-specific densities are optimized such that the expression pattern of predicted markers best matches the experimental marker distribution, for all markers.

The goal is to solve for the cell densities of each morphological type (m-type) in every bin of the neocortical column (Profile Matrix), using as input a Transcript Expression Matrix derived from RT-PCR and a Protein Matrix derived from the stained images. An overview of the process is given in Figure 2.

The “Transcript Expression Matrix” is the measure of the frequency of each RNA transcript marker for each interneuron m-type (data from RT-PCR experiment, Figure 1B). The dimensions of this matrix are the m-types by the measured number of transcript. If every cell of a particular m-type expressed a given RNA marker, the frequency of expression would be 1. If the targeted RNA is not expressed, the frequency of expression is 0. After averaging across m-types, the expression frequency is hence a value between 0 and 1.

The “Protein Matrix” is the measure of the density of cells expressing each protein marker in every bin of the neocortical column (data from the immunohistochemistry experiment, Figure 1A). The dimensions of this matrix are the number of protein-positive cells by the number of bins. It is equal to the product of the proportion of each morphological type expressing the transcript marker and the profile matrix, as in the following equation:

$$\text{[Protein Matrix]}=\begin{array}{c} \text{[Transcript Expression Matrix]}\\ \text{[Profile Matrix]} \end{array}$$

Therefore, the Profile Matrix can be solved for as:

$$\text{[Profile Matrix]}=\begin{array}{c} {\text{[Transcript Expression Matrix]}}^{-1}\\ \text{[Protein Matrix]} \end{array}$$

In order to obtain a better estimate for inhibitory cell type density, we constrained the total sum of inhibitory neurons types to be equal to the total measured distribution of inhibitory interneurons, as measured from GABA staining. This was done by appending a row corresponding to the inhibitory cell density to the protein matrix and appending a corresponding row of ones to the transcript expression matrix. Another constraint was that the solution be non-negative. We could then solve for the profile matrix that would best satisfy the constraints.

In order to obtain a robust prediction of cell-type profiles reflecting the uncertainty in each input measurement, we sampled from a normal distribution with the standard deviations measured for every entry in the transcript expression matrix and the protein matrix. This process was repeated 500 times and the results were averaged together to obtain the final profile matrix used as the solution. We performed this fitting process in Matlab (Mathworks, Natick, MA, USA). Hence, the final output represents an average brain, rather than a unique individual.

The sample size of collected Chandelier cells in the RT-PCR data set was low (*n* = 4 of 226 total cells). Therefore, we did not attempt to fit ChC but rather took their proportion in each bin to be a fixed proportion (1.8%) of the total inhibitory cell density in that bin. All of the other inhibitory cell types were obtained from the predicted profiles.

## Results

### Single-Cell Electrophysiology, Morphology and Transcriptomics

The RT-PCR data set collected in Toledo-Rodriguez et al. (2005) was used. It had 45 MCs, 11 BPs, 27 BCss, 4 ChCs, 12 DBs, 69 LBCs, 47 NBCs, and 11 SBCs Almost all MCs expressed SOM (98%). BPs expressed CR and VIP at moderate frequencies (36% and 45%). BCs expressed CB and CR at moderate frequencies (35% and 33%). DBs expressed VIP at high frequency (73%). SBCs expressed CB at moderate frequencies (36%). LBCs and NBCs expressed most markers except for CR and VIP.

### Immunohistochemistry

#### Neuron Fractions

**Figure 3:**
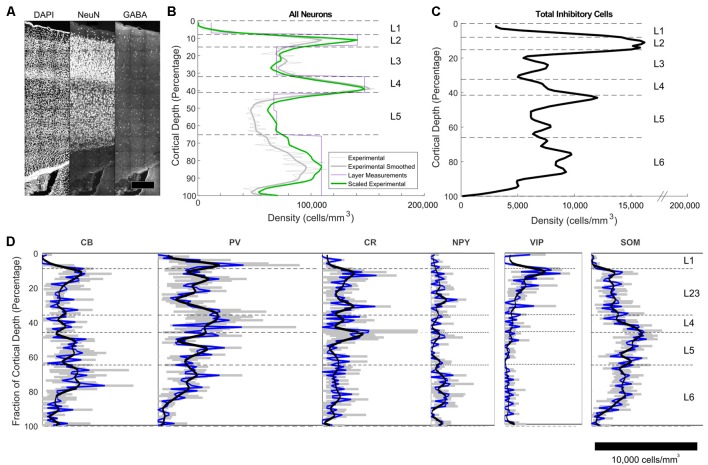
Immunohistochemistry-labeled cell densities across cortical layers. **(A)** NeuN and GABA+ cells were stained and counted in the same slice to obtain density estimates. **(B)** Total neuron density smoothed estimates using 100 bins are shown in the green line. The purple line shows estimates obtained in the center of each layer using stereological techniques (Markram et al., 2015). The green line shows the final scaled version of the neuron estimates. This was obtained by scaling the raw densities to match the more accurate stereologically-obtained layer densities at the center of each layer (Markram et al., 2015). Scaling factors were L1: 2.36, L2: 1.34, L3:0.9, L4:1.04, L5a:1.28, L5b: 1.37, L6: 1.15. **(C)** Total inhibitory interneuron density from the experiment. **(D)** Cell density as a function of cortical depth for common interneuron protein markers: calbindin (CB), calretinin (CR),neuropeptide Y (NPY), parvalbumin (PV), somatostatin (SOM) and vasointestinal peptide (VIP). A cortical depth of zero corresponds to the top of L1, while 100 is the bottom of L6. The blue lines are the averages data, while the black lines are the smoothed data. Gray indicates the range of the standard error of the mean for the average values.

The densities of excitatory and inhibitory neurons per bin (E-I fractions) were established by counting cells stained for NeuN (all neurons), and GABA (all inhibitory neurons) in the tissue block (Figure 3A). For visualization of laminar and area boundaries and for cell counting quality procedures, slice was stained for DAPI. Overall, excitatory and inhibitory neurons represented 87% ± 1% and 13% ± 1% of the population, respectively, with a trend toward higher fractions of excitatory neurons in deeper layers (Figures 3B,C). Total neuron density was highest in L2 and L4 (Figure 3B). Inhibitory cell density was highest in L2 (Figure 3C).

#### Inhibitory Protein Markers

**Figure 4:**
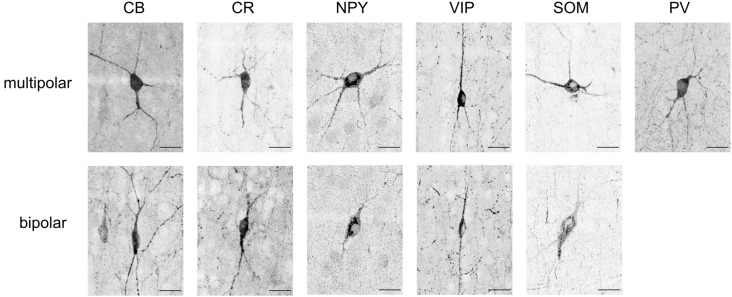
Maximum intensity projections of typical soma shapes observed for various markers (CB, CR, NPY, VIP, SOM, PV). The upper line shows multipolar neurons, whereas the lower line shows bipolar neurons. Note that no example of bipolar-shaped somas could be found for PV. Scale bar is 25 μm.

The IF images produced high-resolution volumetric data sets. At least three brain slices (data sets) were analyzed for each protein marker (Figure 3D), from at least two animals per marker (sources delineated in Supplementary Material). The chosen antibodies targeted epitopes localized in the cell body, allowing counting of cells (Figure 4). The average coefficient of variation per bin was 1.7 across all stains. This high variability might be attributable to the fact that expression levels are rapidly changing at this age (Sánchez et al., 1992; Schierle et al., 1997).

CB positive inhibitory cells (*n* = 3 slices) showed a heterogeneous distribution throughout the cortical column depth (from L2 to L6), exhibited layer-averaged peaks in L2/3 and L5, consistent with previous studies in adult rat (Gonchar and Burkhalter, 1997). With rare cell marked in L1 (4% of labeled cells in volume), a highest number in L2/3, L5 and L6 (29%, 23% and 36% of labeled cells in volume, respectively). As expected (Hof et al., 1999), some pyramidal cell bodies were stained and we observed subcellular localization of the CB stain in cytoplasm and nuclei for both interneurons and pyramidal cells, in addition to arborization stainings. Only pyramidal cells from L2/3 seem to be CB-positive (Staiger et al., 2004; Gonchar et al., 2008). Considering the stains enabled morphological visualization, we also observed different anatomical types, bipolar and multipolar for interneurons, and pyramidal for excitatory cells. The average density across S1HL somatosensory cortex was 2,124 ± 322 cells/mm^3^.

Inhibitory neurons stained for CR (*n* = 3 slices) showed a heterogeneous distribution throughout the cortical column depth, with rare cell marked in L1, L5 and L6 (5%, 19%, and 26% of cells in volume, respectively) and a highest number in L2/3 (36% of cells in volume). The layer-averaged peaks in L2/3 and L5 agree with studies of adult rats (Gonchar and Burkhalter, 1997). We observed subcellular localization of the CR stain in cytoplasm and nuclei for interneurons cells, in addition to arborization stainings. We observed bipolar and multipolar interneurons anatomical types. The average density across S1HL somatosensory cortex was 1,576 ± 554 cells/mm^3^.

Inhibitory neurons stained for SOM (*n* = 5 slices) showed a homogenous distribution throughout the cortical column depth, except in L1 (1% of cells in volume), and a highest density in L5 and L6 (29% and 32% of cells in volume, respectively). We observed subcellular localization of the SOM stain in the cytoplasm (nucleus not stained) for interneurons cells, in addition to arborization stainings (with a highest density in L1). We observed different anatomical types with a majority of the cells being multipolar but some showing a bipolar morphology with ovoid-shaped soma (Wang et al., 2004). SOM peaked in L6, in agreement with the adult rat (Gonchar and Burkhalter, 1997). The average density across S1HL somatosensory cortex was 2,496 ± 344 cells/mm^3^.

PV (*n* = 3): as expected (Tremblay et al., 2016), inhibitory neurons stained for PV showed a heterogeneous distribution throughout the cortical column depth, with lower amount of cells marked in L1 (10% of cells in volume) and a highest number in L4 (15% of cells in volume). We observed subcellular localization of the PV stain in cytoplasm and nuclei for interneurons, in addition to arborization stainings (highest density in L4). PV peaked in L4, in contrast to the adult animal, in which a peak was observed in L5 (Gonchar and Burkhalter, 1997). We observed multipolar PV positive interneurons. The average density across S1HL somatosensory cortex was 3,160 ± 554 cells/mm^3^.

NPY positive inhibitory cells (*n* = 3 slices) showed a heterogeneous distribution throughout the cortical column depth, with rare cell marked in L1 (4% of cells in volume) and a highest number in L5 and L6 (14% and 45% of cells in volume, respectively). These results differ from previous reports of NPY-expressing interneurons as a majority of L1 interneurons (Karagiannis et al., 2009). We observed subcellular localization of the NPY stain in the cytoplasm and arbors (highest density in L1), but not in the nucleus. We observed different anatomical inhibitory cell types: bipolar and multipolar. NPY density peaked in L6. The average density across S1HL somatosensory cortex was 676 ± 244 cells/mm^3^.

As shown in the barrel cortex of mice (Prönneke et al., 2015), inhibitory neurons stained for VIP (*n* = 3 slices) showed a heterogeneous distribution throughout the cortical column depth, with fewer cells marked in L5 and L6 (9% and 14% of cells in volume, respectively) and a higher number in L2/3 (57% of cells in volume). We observed subcellular localization of the VIP stain in the cytoplasm (nucleus not stained), in addition to arborization stainings. Considering the stains enabled morphological visualization, the majority of the positive cells show a fusiform anatomical type and in L5 and L6 a multipolar pattern. The average density across S1HL somatosensory cortex was 797 ± 187 cells/mm^3^.

### Fitting of Cell Types

**Figure 5:**
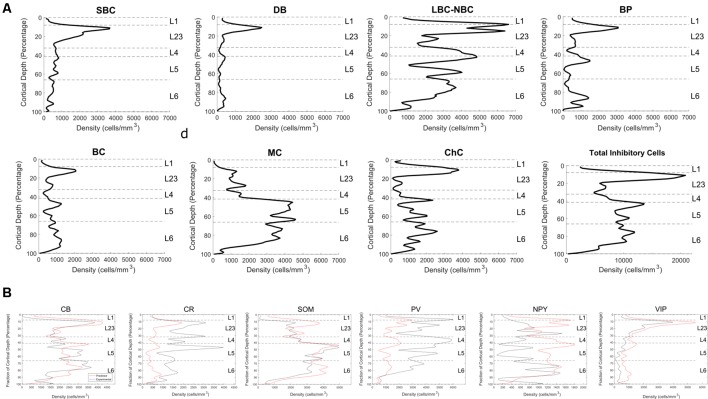
High-resolution fitting results. **(A)** Experimental profiles for Small Basket Cells (SBC), Double Bouquet (DB), Large and Nest Basket Cells (LBC-NBC), Bipolar cells (BP), Bitufted Cells (BC), Martinotti Cells (MC), Chandelier cells (ChC). **(B)** The predicted marker distribution (red) and experimental marker distribution (black).

Applying the fitting procedure (see equations in “Materials and Methods” section and Figure 2 for explanation) produced an estimate of the morphological types within each layer (Figure 5). L1 cell types used were not present in the RT-PCR data set, so we cannot predict these the composition of L1. With this caveat, we performed the mapping of the cell types from the other layers to L1 for the sake of completeness.

With the limited number of markers, LBC and NBC cells cannot be reliably distinguished in the RT-PCR data set. Therefore, for the purposes of fitting, we combined them into a common group, LBC-NBC. After the profile was assigned, it was post-facto re-divided into separate LBC and NBC groups. Accordingly, 59.5% of the LBC-NBC profiles were assigned to be LBC and the rest to NBC, since this proportion reflects the frequency in the sampled dataset.

It is not possible to perfectly match all the original staining profiles. NPY positive cells are relatively few so the error contribution is outweighed by the contribution of the other cell types. PV is expressed primarily by the LBC/ NBC morphology type in the data set, which also happens to express CB, NPY, and CCK at moderate levels. Improved fitting of the PV marker would therefore likely result in poorer match for the other markers. Division of the original morphology classes into additional subtypes could potentially improve the fit.

**Figure 6:**
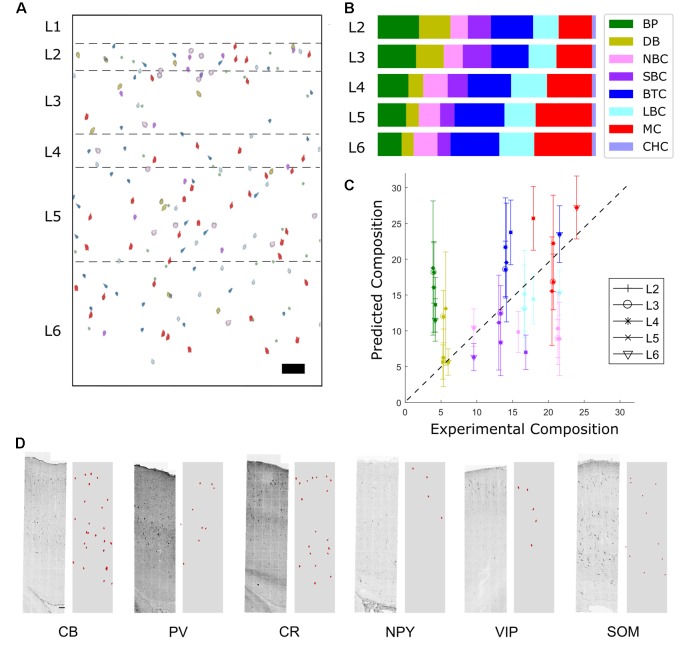
Distribution of predicted marker expression and validation. **(A)** Virtual slice. Scale bar is 100 μm. Note that layer 1 cell types cannot be predicted using the available data and so are not included. **(B)** Composition results. The most prevalent types are Martinotti cells, Large Basket Cells, and Nest Basket cells. **(C)** Correlation of predicted fractions vs. experimental fractions (*r* = 0.85) and experimental comparison of the two most common inhibitory cell types (LBC and MC). In the case of a perfect prediction, all points would lie on a straight line. BPs are overpredicted while NBCs are underpredicted. **(D)** Comparison of predicted expression of protein cell markers (right subpanels, red) to experimental stainings (left). Scale bar is 100 μm.

Predicted densities were used to make a virtual slice plot in which cells of each type were placed in representative layer positions (Figure 6A) and a layer-wise bar plot (Figure 6B). The percent composition in L2/3 is dominated by LBCs and NBCs, while in the deeper layers MCs are also prevalent. Other cell types, most notably SBCs, make up the remainder.

To validate the results, transcript expression density by layer was compared to the result of a previous estimate obtained by large-scale experimental sampling (Markram et al., 2015). Although the most frequent morphological types exhibited acceptable agreement and correlation with the experimental results (Figure 6C), lower frequency expressed morphological types could not be reliably distinguished. This aspect can be explained by high variability of immunohistochemistry and RT-PCR measurement, and can be improved with larger sample size.

Some common trends are apparent when comparing the experimental and predicted profiles of the most frequent cell types (Markram et al., 2004). LBCs and NBCs are broadly expressed, with a peak in percent composition in L4. This is in agreement with results in the literature (ibid.). The predicted MCs cell density proportion peaks in the deep layers, also consistent with literature results (ibid.). SBCs cell density is higher expressed in L2/3, again consistent with literature results (ibid.). Trends for less frequent cell types were not as evident. Some common trends are apparent when comparing the experimental and predicted profiles of the most frequent cell types.

Finally, the expression profile resulting from the cell distribution prediction was compared to the original experimental staining profile (Figures 5B, 6D). Although the contour and trends are generally followed, mismatch in the magnitude occurs.

## Discussion

In this work, we have developed a fitting method for estimating the distribution of inhibitory interneurons in P14 rat somatosensory cortex. This method obviates the need to directly sample cortical regions to determine morphological composition and is, therefore, less laborious than traditional methods.

Every predicted configuration of inhibitory interneuron profiles results in a corresponding total marker expression profile when cells expressing a given marker are summed. The method finds the density of cells of all types that minimizes the error between the predicted expression of markers and the experimental immunostained images of the same markers.

This method offers several advantages over traditional methods: it is less prone to bias caused by certain cell types being chosen over others in the sampling process; this is because it uses cell density information collected by counting cell somas directly and not staining intensity as a proxy for cell density as done in other methods (Grange et al., 2014), it is not subject to artifacts caused by variations in soma sizes. However, we recognize that there could be some cell counting bias in subjectively determining if a cell positively expresses a particular marker.

Finally, it uses cell types that have been linked to both morphology and electrophysiology, allowing the use of an extensive dataset collected for other purposes (Toledo-Rodriguez et al., 2005). The working assumption is that levels of RNA expression for a particular gene correspond well with the protein expression obtained *via* immunohistochemistry. This may not necessarily be the case since protein degradation rates can differ and translational regulatory mechanisms can result in different levels of protein relative to mRNA levels.

One drawback of the method is that, because it uses image data drawn from different slices, the fit profiles do not necessarily correspond to any particular individual cell. Recent advances in colabeling of the transcriptomic state of single cells (Codeluppi et al., 2018; Wang et al., 2018) have the potential to allow more accurate assignment of cell types.

For the most part, the results obtained by the new method agree with the trends seen in earlier work in which hundreds of cells were sampled in different layers (Markram et al., 2004, 2015). The calculated MCs density in the deep layers in our prediction is lower than these estimates. Furthermore, we have additional substructure in the distributions due to the finer sampling bins used. This substructure is most evident at the layer 1–layer 2 boundary. The overall inhibitory cell profile also agrees with experimental measurements (Meyer et al., 2011).

Two factors, in particular, could lead to improve results. First, there are likely to be more subdivisions of inhibitory cell types than the ones used here. For example, transcriptome-based study described 16 types of interneuron in the somatosensory cortex (Zeisel et al., 2015). Subtypes can be expected to exhibit different properties in electrical behavior. Furthermore, we expect that additional transcriptomics data will also allow improved subtype classification of neurons and identification of markers specific to those types. In this manner, the approach can be extended to include more cell types and additional regions of the brain. If more subtypes were to be mapped, however, one would need to use more protein markers for different cell-specific gene products. Second, the accuracy of the results would benefit from more antibody staining image replicates for each marker. As automatic cell counting methods improve, more cell counting datasets suitable for this method will become available. Since the mean cell density for a given marker is used as input in the process, having more replicates would decrease the standard error of the mean (increase the certainty of estimation of the true mean).

Overall, we have shown that a fitting approach can be used to estimate cell densities at finer levels of resolution than previously possible. Cell type distributions are predicted without the need to count each type separately. The method is scalable to accommodate more data as it becomes available.

## Data Availability

All datasets generated for this study are included in the manuscript and/or the Supplementary Files.

## Ethics Statement

The animal study was reviewed and approved by Veterinary Authorities and the Cantonal Commission for Animal Experimentation of the Canton of Vaud, according to the Swiss animal protection laws.

## Author Contributions

DK and HM designed the experiments. JM performed immunohistochemistry experiments, cell counting and post-processing immunohistochemistry data analysis. RG and OB performed image-processing analysis. DK and RV performed RT-PCR analysis. DK designed and performed the fitting analysis. DK, JM, FS, and HM wrote the manuscript.

## Conflict of Interest Statement

The authors declare that the research was conducted in the absence of any commercial or financial relationships that could be construed as a potential conflict of interest.

## Footnotes

^1^https://imagej.nih.gov/ij/plugins/cell-counter.html
