# Erratum: A Derived Positional Mapping of Inhibitory Subtypes in the Somatosensory Cortex

**DOI:** 10.3389/fnana.2019.00097

**Published:** 2019-12-11

**Authors:** Frontiers Production Office

**Affiliations:** Frontiers Media SA, Lausanne, Switzerland

**Keywords:** cell density, rat brain, inhibitory interneurons, somatosensory cortex, composition, cell types, cell counting, neuronal distribution

Due to a production error, the final version of the Supplementary Table 1 file was not updated in the published version of the article.

The publisher apologizes for this mistake. The original article has been updated.

